# Leveraging Eye Tracking to Prioritize Relevant Medical Record Data: Comparative Machine Learning Study

**DOI:** 10.2196/15876

**Published:** 2020-04-02

**Authors:** Andrew J King, Gregory F Cooper, Gilles Clermont, Harry Hochheiser, Milos Hauskrecht, Dean F Sittig, Shyam Visweswaran

**Affiliations:** 1 Department of Biomedical Informatics University of Pittsburgh Pittsburgh, PA United States; 2 Department of Critical Care Medicine University of Pittsburgh Pittsburgh, PA United States; 3 Intelligent Systems Program University of Pittsburgh Pittsburgh, PA United States; 4 Department of Computer Science University of Pittsburgh Pittsburgh, PA United States; 5 Department of Biomedical Informatics University of Texas Health Science Center at Houston Houston, TX United States

**Keywords:** electronic medical record system, eye tracking, machine learning, intensive care unit, information-seeking behavior

## Abstract

**Background:**

Electronic medical record (EMR) systems capture large amounts of data per patient and present that data to physicians with little prioritization. Without prioritization, physicians must mentally identify and collate relevant data, an activity that can lead to cognitive overload. To mitigate cognitive overload, a Learning EMR (LEMR) system prioritizes the display of relevant medical record data. Relevant data are those that are pertinent to a context—defined as the combination of the user, clinical task, and patient case. To determine which data are relevant in a specific context, a LEMR system uses supervised machine learning models of physician information-seeking behavior. Since obtaining information-seeking behavior data via manual annotation is slow and expensive, automatic methods for capturing such data are needed.

**Objective:**

The goal of the research was to propose and evaluate eye tracking as a high-throughput method to automatically acquire physician information-seeking behavior useful for training models for a LEMR system.

**Methods:**

Critical care medicine physicians reviewed intensive care unit patient cases in an EMR interface developed for the study. Participants manually identified patient data that were relevant in the context of a clinical task: preparing a patient summary to present at morning rounds. We used eye tracking to capture each physician’s gaze dwell time on each data item (eg, blood glucose measurements). Manual annotations and gaze dwell times were used to define target variables for developing supervised machine learning models of physician information-seeking behavior. We compared the performance of manual selection and gaze-derived models on an independent set of patient cases.

**Results:**

A total of 68 pairs of manual selection and gaze-derived machine learning models were developed from training data and evaluated on an independent evaluation data set. A paired Wilcoxon signed-rank test showed similar performance of manual selection and gaze-derived models on area under the receiver operating characteristic curve (*P*=.40).

**Conclusions:**

We used eye tracking to automatically capture physician information-seeking behavior and used it to train models for a LEMR system. The models that were trained using eye tracking performed like models that were trained using manual annotations. These results support further development of eye tracking as a high-throughput method for training clinical decision support systems that prioritize the display of relevant medical record data.

## Introduction

### Background

Electronic medical record (EMR) systems capture considerable amounts of patient data, especially in data-rich care settings like intensive care units (ICUs) [1]. The large amount of data per patient necessitates that the user interfaces of EMR systems help users rapidly understand the medical state of each patient [2]. However, patient data in EMR systems are typically presented to physicians with little prioritization, leading to the risk of cognitive overload, which in turn may lead to reductions in physician ability to identify important patient data for clinical assessment and management and increases in risks of medical error [2]. One approach to overcoming this drawback envisions an adaptive EMR system that draws the physician’s attention to the right data, for the right patient, at the right time, and in the right way [3]. As a step toward this goal, we developed a Learning EMR (LEMR, pronounced lemur) system that uses physician information-seeking behavior to prioritize the display of relevant medical record data [4]. Our system relies on supervised machine learning models that predict which patient data are likely to be useful in a specific context, where the context refers to a particular combination of clinical user, clinical task, and patient case [5]. The models will be trained on thousands of observations of patient data items that different physicians have sought as relevant across a wide range of patient cases and clinical tasks.

The acquisition of training data is a critical barrier to the development of a LEMR system. Some observations of physician information-seeking behavior are recorded in commercial EMR systems through the capture of mouse clicks, but such data on user behavior are often captured with insufficient resolution for training a LEMR system. Another method for collecting behavior data is through manual annotation of relevant data by clinical users. In a prior study, we collected manual annotations from physicians who reviewed patient cases and identified which data were relevant for a simulated clinical task [5]. With this data, we developed machine learning models that enable the LEMR system to identify likely relevant patient data in similar clinical contexts. However, manual annotation is disruptive and time-consuming, and thus, it limits the amount of training data that can be collected. To have a broad coverage of clinical contexts, larger amounts of training data are needed for model construction. Moreover, while manual annotation is possible in a research setting, it is infeasible in routine clinical practice where it would dramatically increase the clinical workload. Eye tracking offers a method that can potentially capture information-seeking behavior unobtrusively as a byproduct of care delivery [6]. In this paper, we investigated the use of eye tracking as an automated high-throughput method to capture physician information-seeking behavior.

### Related Work

The use of eye tracking in studies of EMR systems has focused on understanding users and their interactions with systems. Investigators have used eye tracking to understand clinical reasoning [7], discern information search patterns [8], measure cognitive loads while performing tasks in the EMR system [9], evaluate usability [10], and measure time use [11]. Moacdieh et al [12] used eye tracking to demonstrate that display of irrelevant data increases cognitive workload, and Gold et al [13] showed that a commercial eye-tracking device can be used to assess an EMR system’s usability.

We are not aware of any studies that have used eye tracking to train machine learning models of information-seeking behavior in an EMR system. In previous work, we established the feasibility of using a low-cost eye tracker to identify items of interest on an EMR display [14]. We conjectured that the resulting eye-tracking data can be used to infer which medical record data are viewed by physicians. Such data can then be used to train machine learning models that the LEMR system would use to identify medical record data in a future patient case that are likely to be relevant to the user. Moreover, the availability of inexpensive, portable eye-tracking devices could make broad deployment of such systems feasible.

In this paper, we propose and evaluate a novel approach to using eye tracking to capture information-seeking behavior that can subsequently be used to build models that identify relevant patient data in a LEMR system. We compare the performance of supervised machine learning models trained on eye tracking–derived annotations with that of models trained on manually obtained annotations. We hypothesize that eye tracking is an effective, high-throughput alternative to manually annotating training data for the LEMR system.

### Learning Electronic Medical Record System

**Figure 1:**
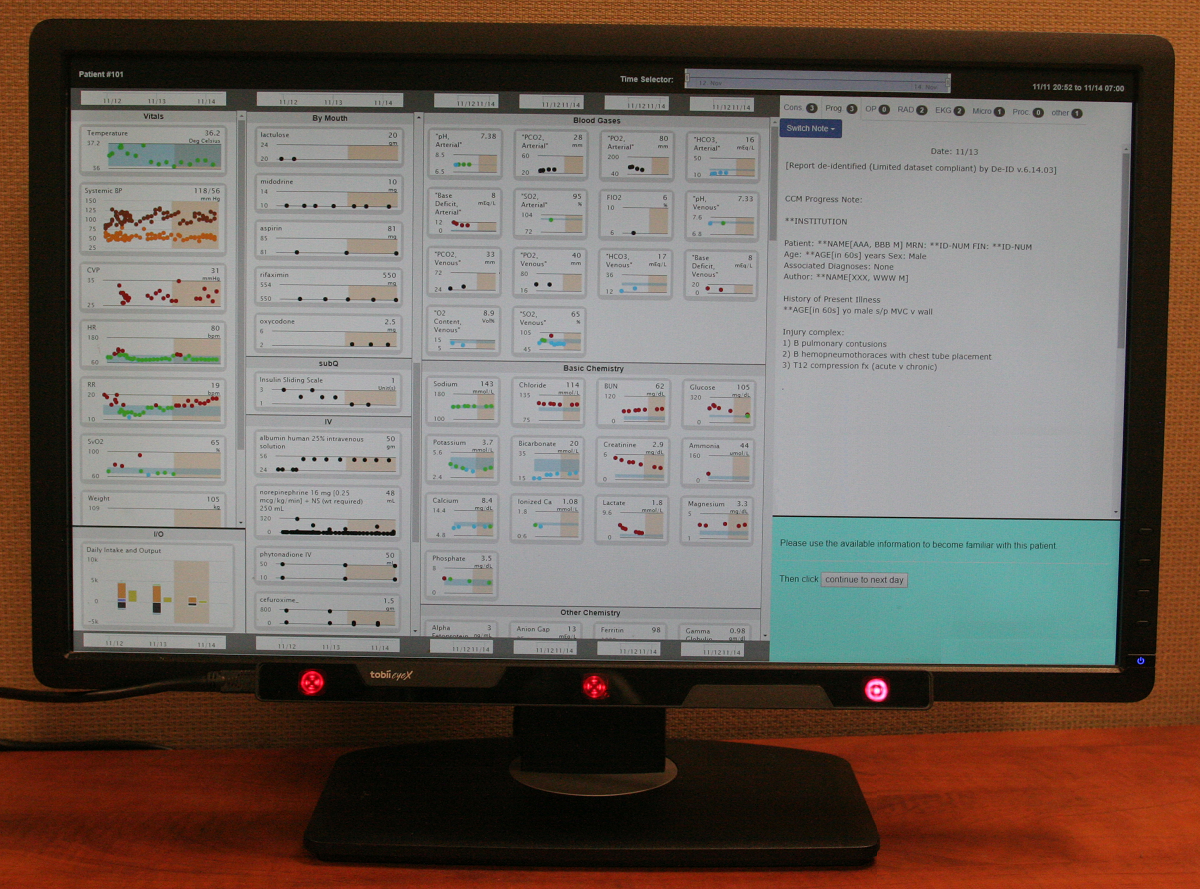
A computer monitor displaying the Learning Electronic Medical Record user interface with an eye-tracking device mounted at the bottom edge of the monitor. The interface temporally displays patient medical record data in four scrollable panels: (from left to right) panel 1 contains vital signs, ventilator settings, and intake and output; panel 2 contains medication administrations; panel 3 contains laboratory test results; and panel 4 contains free-text notes and reports. The remote eye-tracking device is magnetically attached to the bottom edge of the monitor and connected to the computer via a universal serial bus cable (screenshot is of a deidentified patient case).

A LEMR system prioritizes the display of medical record data based on predicted relevance [4]. To learn relevance, our prototype LEMR system enables the collection of physician information-seeking behavior. We used two collection methods in this study: (1) manual selection, where the user annotates relevant medical record data by clicking checkboxes displayed on the user interface, and (2) gaze-derived, where an eye-tracking device captures eye gaze patterns while the user is reviewing a patient’s medical record. Figure 1 shows the LEMR system’s user interface on an eye tracking–equipped computer monitor. The software for the LEMR user interface is available on GitHub [15].

## Methods

### Eye Tracking

We used the Tobii EyeX (Tobii Gaming) eye-tracking device, an inexpensive portable eye-tracking device and software package marketed for computer gaming and virtual reality applications [16] (as of publication, EyeX’s successor product is called Tobii Eye Tracker 4C). The EyeX device samples eye gaze coordinates on the monitor at approximately 60 Hz. To operate the device and read the stream of gaze data, we used open-source Python bindings for the Tobii Gaze Software Development Kit [17].

A recent evaluation of the Tobii EyeX found that for many research applications temporal and spatial resolutions are modest, precision is moderate, and sampling frequency is low [18]. Despite the EyeX’s limitations, the authors noted that it is adequate for applications that do not require more than monitoring of simple eye movements. For example, the EyeX would not be suitable for measuring every saccade and fixation of a person reading a paragraph of text but is suitable for determining if a region of the monitor was gazed upon. In a prior study, we found the EyeX to be comparable in performance to a more expensive, research-grade eye-tracking device for capturing information-seeking behavior while using the LEMR user interface (the difference in errors of the two devices was less than a predefined noninferior margin of 11 pixels at the 95% confidence interval) [14].

The eye-tracking device provides time-stamped gaze position coordinates on the computer monitor but does not provide concomitant information on data displayed on the monitor. Therefore, to use eye tracking to automatically capture physician information-seeking behavior, the stream of gaze position coordinates must be mapped onto elements of the user interface. Because the LEMR user interface is dynamic (it changes as users scroll through the medical record data), we separately record a stream of interface element locations on the monitor that can be mapped to gaze position coordinates obtained from eye tracking. Each interface element contains one medical record data item (eg, the time series of blood glucose measurements). Using time-stamps in the two data streams, gaze position coordinates are translated to medical record data items. A data item such as blood glucose measurements with a gaze dwell time that is longer than a set threshold (eg, 250 milliseconds) is designated as a positive training sample for that data item. Positive and negative training samples with respect to a particular data item denote that the data item was viewed and was not viewed by a user, respectively. We developed and implemented an algorithm that rapidly processed concomitantly acquired gaze position coordinates and interface element locations. This algorithm is described in our prior work [14]. The scripts for eye tracking are available on GitHub [19].

### Data Collection

#### Overview

For machine learning, we created two training data sets of physician information-seeking behavior. One data set consisted of targets obtained from manual user selection; the second consisted of targets obtained from gaze position coordinates captured by eye tracking. We created an independent evaluation data set using manual annotation. Reviewers were presented with both structured EMR data (eg, demographics, diagnosis, vital sign measurements, ventilator settings, laboratory test results, medication administrations, procedures, microbiology culture results, and intake and output) and free text data (eg, admission notes, radiology reports) when reviewing a case. However, only structured EMR data were used for machine learning.

#### Reviewers

We recruited critical care medicine physicians from the University of Pittsburgh’s Department of Critical Care Medicine to review and annotate patient cases: 11 physicians participated in the training phase and 12 physicians participated in the evaluation phase. Five reviewers who participated in the training phase also participated in the evaluation phase. Details of characteristics of the reviewers are shown in Table 1. Every reviewer used the LEMR system (shown in Figure 1). All reviewers were able to familiarize themselves with the interface by reviewing several practice cases before data collection began.

**Table 1:** Characteristics of reviewers.

Phase of study	Number of reviewers	Time in years since medical school graduation, mean (SD)	Time in years spent in ICU^a^, mean (SD)	Weeks per year spent rounding in the ICU, mean (SD)
Training	11	5.3 (3.0-10.0)	1.8 (0.3-7.0)	34 (26-42)
Evaluation	12	5.4 (3.0-11.0)	1.7 (0.6-4.0)	36 (28-44)

^a^ICU: intensive care unit.

#### Simulated Clinical Task

Reviewers were asked to use the LEMR interface to conduct prerounding, which involves the review of a patient’s medical record and identification of relevant data for a summary presentation at morning rounds. Each patient case was loaded into the LEMR system and shown to a physician reviewer (an example patient case is shown in Figure 1). The physician reviewed the case and completed three tasks: familiarization, preparation, and selection.

In the familiarization task, the physician was shown the patient’s medical record data from hospital admission until 8:00 am on a random ICU day during the ICU stay between day 2 and the day before discharge from the ICU. The physician was instructed to review the data and become familiar with the case as if it were one of their patients.

In the preparation task, the physician was shown an additional 24 hours of the patient’s medical record data and instructed to review the data and prepare to present the case during morning rounds. During this task, we used eye tracking to record the physician’s gaze position coordinates from which we inferred the physician’s information-seeking behavior.

In the selection task, a checkbox was added to each user interface element containing a medical record data item (such as blood glucose measurements). The physician manually annotated (by clicking on checkboxes) data items they considered to be relevant for the task of prerounding.

#### Patient Cases

For the creation of training data sets, 178 patient cases were randomly selected from patients who were admitted to an ICU between June 1, 2010, and April 30, 2012, at the University of Pittsburgh Medical Center (PA). For the creation of the evaluation data set, 18 patient cases were randomly selected from patients admitted to an ICU between June 1, 2012, and December 31, 2012. Each selected patient had a diagnosis of either acute kidney failure (AKF; ICD-9 584.9 or 584.5) or acute respiratory failure (ARF; ICD-9 518.81). The number of patients with each diagnosis was roughly equal in the training and evaluation data sets. EMR data for the patients were extracted from a research database and deidentified to remove all unique identifiers except for dates and times related to events.

#### Annotations

We created two training data sets that contained the same patient cases but differed in the construction of the targets. In the manual selection training data set, targets were derived from the checkbox clicks recorded during the selection task; in the gaze-derived training data set, targets were derived from eye tracking during the preparation task. From the 178 patient cases reviewed, 44 cases were discarded either because the eye-tracking data were incomplete (which occurred when a reviewer’s head was not within the trackable range of the eye-tracking device) or the selection task was skipped inadvertently. Thus, the two training data sets consisted of the same 134 patient cases. Note that each patient case was reviewed by a single reviewer to maximize the number of patient cases that could be reviewed with a limited number of physicians. For the evaluation data set, 18 patient cases were each reviewed by 4 physicians for a total of 72 patient cases. Four cases were discarded because a reviewer inadvertently skipped the annotation task, and thus the evaluation data set contained 68 patient cases with manually annotated targets.

### Machine Learning

#### Problem Description

The LEMR system prioritizes the display of relevant medical record data. To determine which data are relevant in a specific context (a combination of user, task, and patient), the LEMR system uses supervised machine learning models. For this study, we focused on the information-seeking differences among patient cases when the user and task were constant (all users were critical care physicians who performed the same task of prerounding). The training data sets consisted of a large number of predictor and target variables.

#### Predictor Variables and Feature Construction

The patient data we used as predictor variables included simple atemporal variables such as demographics (6) and diagnosis (1) and complex variables representing time series, including vital sign measurements (14), ventilator settings (9), laboratory test results (814), medication administrations (1207), procedures (394), microbiology culture results (10), and fluid intake and output (7). We derived a fixed set of features from each complex variable so that standard machine learning methods could be applied readily. For example, for a time-stamped sequence of serum glucose levels, we generated 35 features that include the first glucose value during the ICU stay, most recent value, highest and lowest values until the current time, difference between the most recent two values, and 30 other features [20]. Thus, we generated 35 features for each laboratory test and each vital sign, 31 features for each ventilator setting, 9 for each medication administration, 4 for each culture and each procedure, and 2 for each intake and output variable. In summary, a patient case was represented by a fixed-size vector of 13,596 features that summarized the clinical evolution of the patient’s condition from the time of admission to the ICU to the current time.

#### Target Variables

We represent physician information-seeking behavior with a set of binary target variables. A target variable is created for any medical record data item that a physician could seek as relevant. For a specific patient case, a data item’s corresponding target variable indicates if the item was sought as relevant or not (eg, the glucose target variable is positive if the physician sought the serum glucose levels). We derived two sets of targets; one set of targets was derived from manual selections and another from gaze position coordinates. In the manual selection training data set, a target variable, such as glucose target, was assigned the value positive if the physician selected the checkbox that was displayed with the data item; and a target was assigned the value negative if the associated checkbox was not selected. In the gaze-derived training data set, a target was assigned the value positive if the physician gazed at that item for at least 250 milliseconds and was assigned the value negative if the physician gazed at it for less than 250 milliseconds. (This threshold corresponds to the average fixation time while a person is reading [21]). In a patient case where a data item was not available (eg, serum glucose levels were not measured), the corresponding target was deemed to be absent, and the case was excluded from the training data used to train a model to predict the relevance of that data item.

#### Preprocessing of Training Data

We applied several preprocessing steps to the training data sets. A feature was removed if it had the same value in all cases. If two or more features had identical values for every case, only one of those features was retained. For example, the binary feature “ever measured” often had the same values across all cases for one or more laboratory tests that are part of a single panel; this occurred because such tests are ordered together. Missing predictor values were imputed using two different methods. In the first method, features were imputed with the median (nominal features were imputed with the mode). In the second method, continuous features were imputed with linear regression, and discrete features were imputed with logistic regression. The two imputation methods resulted in two distinct data sets for the manual selection and gaze-derived targets, respectively (a median-imputed data set and a regression- imputed data set).

To reduce feature dimensionality, we applied a feature selection algorithm that independently considered each predictor variable’s set of constructed features. If a predictor variable’s constructed feature set was predictive of a target (ie, cross-validated area under the receiver operating characteristic curve [AUROC] was greater than 0.55), the feature set was kept in the training set for the model corresponding to that target variable. Otherwise, the feature set was discarded from the training set for the model corresponding to that target variable.

#### Model Training

We applied three classification methods (scikit-learn implementations of L2-penalized logistic regression, support vector classifier, and random forest classifier [22]) to the manual selection and gaze-derived training data sets to develop two categories of models. Within each category, we used cross-validation to select the best performing combination of imputation method and classification method for each target. The best performing combination was then used to train a model from the full training data set. The scripts for feature construction [23], imputation of missing values [24], feature selection [25], and for training and evaluating models [26] are available on GitHub.

## Results

Table 2 provides a summary of the data sets, models trained, and models evaluated. The gaze-derived data set had more positive targets on average, resulting in more targets (ie, data items) having enough training data (>3 positive samples) for model construction. In total, 87 manual selection and 115 gaze-derived models were trained. For 68 targets, models were derived from both data sets. These 68 pairs of models were used in the evaluation, and by coincidence the evaluation data set consisted of 68 patient cases.

**Table 2:** Summary of data sets, models trained, and models evaluated. Each model predicts if a single electronic medical record data item is relevant.

Data set	Counts		
	Number of patient cases	Number of models trained	Number of models evaluated (using the manual-selection evaluation data set)
Manual selection training	134	87	68
Gaze-derived training	134	115	68
Manual selection evaluation	68	—	—

Table 3 provides a summary of the models selected for evaluation. Based on the distribution of models (ie, the number of models columns), random forests and median imputation tended to have higher cross-fold AUROC values on the training set than the other machine learning and imputation methods, respectively.

**Figure 2:**
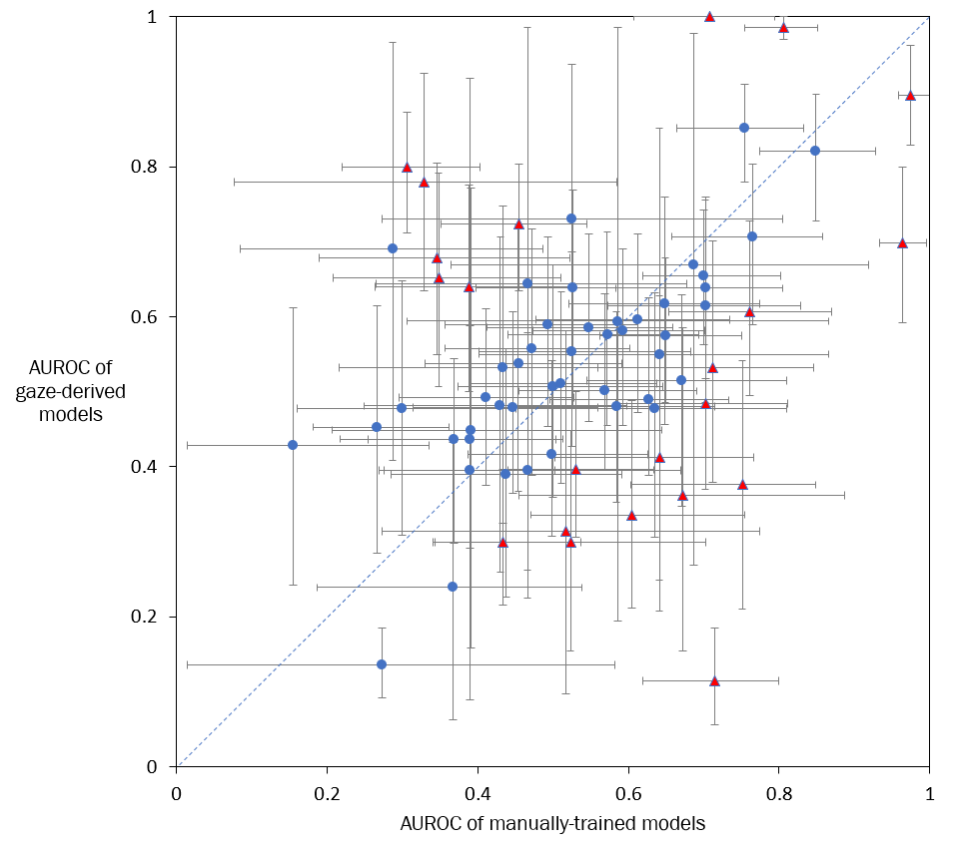
Performance of models used to predict the relevance of targets. The x-axis value and y-axis value of each point indicate the area under the receiver operating characteristic curve (AUROC) values of a pair of manual selection and gaze-derived models, respectively. The vertical and horizontal lines indicate 95% confidence intervals of the AUROC values. The diagonal line indicates equal performance between manual selection and gaze-derived models. Red triangles indicate model pairs where the AUROC value of one model is significantly different than that of the other model (α=.05).

The AUROCs of the 68 pairs of models applied to the evaluation data set are plotted in Figure 2 and shown in Table 4. In Figure 2, points below the diagonal line indicate that manual selection models perform better, and points above the diagonal line indicate that gaze-derived models perform better. The best performing pair of models was for the target alanine aminotransferase (AUROCs of 0.97 for manual selection and 0.90 for gaze-derived), and the worst performing pair was for the target mean corpuscular volume (AUROCs of 0.27 for manual selection and 0.14 for gaze-derived). Statistical differences between each pair of models were calculated from the 95% confidence interval of the average difference in AUROC values using bootstrapping. In 14 instances, the manual selection models performed statistically significantly better, and in 8 instances, the gaze-derived model performed statistically significantly better (see the red triangles in Figure 2 and footnote in Table 4). In the remaining 46 instances, there was no statistically significant difference in AUROCs between the two models (α=.05).

On the Wilcoxon signed-rank test (R software, wilcox.test function), the AUROC values of the manual selection models were similar to the values of the gaze-derived models (*P*=.40). The Wilcoxon signed-rank test is a nonparametric statistical test for comparing two groups of continuous measures.

**Table 3:** Summary of machine learning methods, imputation methods, number of models of each combination of machine learning and imputation methods, and number of features per model.

Machine learning and imputation method		Manual selection		Gaze-derived	
		Number of models	Features per model mean	Number of models	Features per model mean
**Logistic regression**					
	Median	18	207.4	24	176.3
	Regression	10	659.2	15	304.9
**Support vector classifier**					
	Median	18	2366.3	9	2108.9
	Regression	0	—	0	—
**Random forests**					
	Median	27	336.0	37	341.9
	Regression	14	806.1	30	613.8

**Table 4:** Area under the receiver operating characteristic curve values (with 95% confidence intervals) on the evaluation data set of manual selection and gaze-derived models for predicting relevance of targets. Rows are sorted by manual selection performance.

Target	Number of positive samples in evaluation data set	Manual selection models	Gaze-derived models
Alanine aminotransferase	10	0.97^a^ (1.00, 0.96)	0.90 (0.96, 0.83)
Aspartate aminotransferase	10	0.96^a^ (1.00, 0.93)	0.70 (0.80, 0.59)
Norepinephrine	13	0.85 (0.93, 0.77)	0.82 (0.90, 0.73)
Levothyroxine	1	0.81 (0.85, 0.75)	0.99^a^ (1.00, 0.97)
Fraction of inspired oxygen	27	0.77 (0.86, 0.66)	0.71 (0.80, 0.59)
Vancomycin	17	0.76^a^ (0.87, 0.65)	0.61 (0.73, 0.50)
Total bilirubin	12	0.75^a^ (0.85, 0.60)	0.38 (0.54, 0.21)
Bicarbonate, arterial	1	0.75 (0.83, 0.66)	0.85 (0.91, 0.78)
Aspirin	3	0.72^a^ (0.80, 0.62)	0.12 (0.19, 0.06)
pH	6	0.71^a^ (0.85, 0.56)	0.53 (0.70, 0.38)
Troponin	3	0.71 (0.79, 0.61)	1.00^a^ (1.00, 1.00)
Lactate	11	0.70 (0.83, 0.57)	0.62 (0.76, 0.49)
Temperature	42	0.70^a^ (0.81, 0.58)	0.48 (0.61, 0.37)
Lorazepam	3	0.70 (0.80, 0.62)	0.65 (0.74, 0.56)
Ventilator mode	20	0.70 (0.80, 0.58)	0.64 (0.76, 0.52)
Dextrose in water	3	0.69 (0.92, 0.36)	0.67 (0.98, 0.27)
Partial pressure of oxygen	6	0.67^a^ (0.89, 0.45)	0.36 (0.59, 0.15)
Bilirubin direct	9	0.67 (0.81, 0.55)	0.52 (0.63, 0.35)
Heparin	16	0.65 (0.77, 0.52)	0.62 (0.76, 0.46)
Ionized calcium	1	0.65 (0.75, 0.56)	0.58 (0.68, 0.49)
Propofol	6	0.64 (0.87, 0.40)	0.55 (0.85, 0.25)
Piperacillin-tazobactam	15	0.64 (0.81, 0.45)	0.48 (0.63, 0.31)
Famotidine	6	0.64^a^ (0.77, 0.50)	0.41 (0.63, 0.21)
Potassium	26	0.63 (0.73, 0.50)	0.49 (0.63, 0.39)
White blood cells	50	0.61 (0.74, 0.48)	0.60 (0.71, 0.47)
Bands	15	0.60^a^ (0.75, 0.47)	0.34 (0.49, 0.21)
Vancomycin trough	4	0.59 (0.87, 0.31)	0.59 (0.99, 0.19)
Blood urea nitrogen	30	0.59 (0.70, 0.47)	0.58 (0.69, 0.46)
Intravenous base solution	19	0.58 (0.72, 0.45)	0.48 (0.61, 0.35)
Oxygen saturation	29	0.57 (0.69, 0.46)	0.50 (0.63, 0.40)
Intake and output	43	0.57 (0.69, 0.44)	0.58 (0.71, 0.46)
Heart rate	42	0.55 (0.66, 0.41)	0.59 (0.71, 0.46)
Prothrombin time	4	0.53 (0.81, 0.27)	0.73 (0.94, 0.48)
Pantoprazole	9	0.53 (0.71, 0.40)	0.64 (0.77, 0.48)
Bicarbonate, venous	15	0.53 (0.68, 0.40)	0.55 (0.69, 0.43)
Glomerular filtration rate	1	0.53^a^ (0.63, 0.44)	0.40 (0.50, 0.31)
Hydrocortisone	5	0.52^a^ (0.77, 0.27)	0.31 (0.58, 0.10)
Metoprolol	7	0.52^a^ (0.70, 0.34)	0.30 (0.45, 0.16)
Glucose	21	0.51 (0.64, 0.39)	0.51 (0.63, 0.38)
Insulin	13	0.50 (0.65, 0.37)	0.51 (0.67, 0.36)
Platelets	30	0.50 (0.63, 0.39)	0.42 (0.54, 0.31)
Respiratory rate	17	0.49 (0.61, 0.36)	0.59 (0.71, 0.45)
Central venous pressure	2	0.47 (0.68, 0.27)	0.64 (0.99, 0.26)
Creatinine	59	0.47 (0.67, 0.28)	0.40 (0.58, 0.23)
Magnesium	9	0.47 (0.60, 0.36)	0.56 (0.72, 0.39)
Albumin	1	0.46 (0.55, 0.35)	0.72^a^ (0.80, 0.63)
Phosphate	12	0.45 (0.59, 0.33)	0.54 (0.72, 0.37)
Sodium	41	0.45 (0.56, 0.31)	0.48 (0.61, 0.36)
Chloride	12	0.44 (0.59, 0.29)	0.39 (0.56, 0.23)
Blood pressure	61	0.43 (0.64, 0.22)	0.53 (0.75, 0.33)
Chlorhexidine topical	6	0.43 (0.60, 0.25)	0.48 (0.71, 0.26)
Partial thromboplastin time	1	0.43^a^ (0.54, 0.34)	0.30 (0.40, 0.22)
Hemoglobin	31	0.41 (0.53, 0.30)	0.49 (0.61, 0.38)
Neutrophils	4	0.39 (0.64, 0.21)	0.45 (0.77, 0.16)
Fentanyl	14	0.39 (0.53, 0.26)	0.64^a^ (0.78, 0.50)
Metronidazole	13	0.39 (0.51, 0.22)	0.44 (0.59, 0.29)
Furosemide	3	0.39 (0.50, 0.27)	0.40 (0.92, 0.09)
Calcium	2	0.37 (0.54, 0.19)	0.24 (0.43, 0.06)
Sodium chloride	28	0.37 (0.50, 0.25)	0.44 (0.54, 0.30)
International normalized ratio	11	0.35 (0.52, 0.19)	0.68^a^ (0.81, 0.55)
Midazolam	2	0.35 (0.51, 0.21)	0.65^a^ (0.79, 0.51)
Alkaline phosphatase	5	0.33 (0.58, 0.08)	0.78^a^ (0.93, 0.64)
Acetaminophen	1	0.31 (0.40, 0.22)	0.80^a^ (0.87, 0.71)
Ventilator tube status	9	0.30 (0.45, 0.16)	0.48 (0.65, 0.31)
Albuterol-ipratropium	5	0.29 (0.49, 0.09)	0.69 (0.97, 0.41)
Mean corpuscular volume	2	0.27 (0.58, 0.02)	0.14 (0.19, 0.09)
Partial pressure of carbon dioxide	4	0.27 (0.36, 0.18)	0.45 (0.61, 0.29)
Ventilator status	2	0.16 (0.34, 0.02)	0.43 (0.61, 0.24)

^a^Indicates statistically significant difference at α=.05.

## Discussion

### Principal Findings

Current EMR systems capture large amounts of patient data; however, they generally do not capitalize on the opportunity to prioritize display of data in a relevant and context-sensitive fashion. We proposed an intelligent method to identify, display, and focus user attention on relevant medical record data. The rate-limiting step while developing a prototype LEMR system has been the collection of training data. Instead of relying on manual annotations, we proposed using eye tracking as an automated, high-throughput method for capturing physician information-seeking behavior. LEMR models trained on gaze-derived target data performed similarly to models trained on manual selection target data.

### Eye Tracking in Health Care: Pros and Cons

Eye tracking is an alluring method for collecting LEMR training data. First, the availability of inexpensive eye tracking devices makes broad implementation of the devices feasible. With devices installed on many of a hospital’s computer monitors, vast amounts of training data could be recorded. Locally and continuously recorded data could be used to train a LEMR system that is specific to that location’s standards of practice and adaptive to any changes in practice patterns that occur over time. Second, eye tracking is less disruptive to workflow than requesting that physicians rate or annotate the EMR data they are viewing. Finally, eye tracking is more advantageous than manual annotation because it measures what physicians view rather than relying on what they perceive to be relevant, which, if collected retrospectively, could be more affected by recall and attention biases.

Despite its advantages, eye tracking has some drawbacks when used to capture information-seeking behavior. First, to record behavior, the user’s eyes must be within the trackable range (ie, tracking box) of the eye-tracking device. Incorrect positioning led to some data loss during this study and could be exaggerated in a clinical setting where users are often standing or talking with colleagues. Second, to map physician gaze coordinates onto the EMR data shown on the monitor, a stream of the information layout is also needed. We instrumented the EMR interface used in this study to store a stream of the information layout. Adding the same instrumentation to a vendor-based EMR system may be technically feasible but administratively challenging. We are exploring this possibility and other alternatives, such as continuously recording what is on the monitor and parsing the recording using optical character recognition. Finally, we are assuming that a physician views the information they perceive to be relevant, which may not always be the case. More work is needed to determine in which situations gaze patterns are a reasonable approximation for the information physicians seek.

### Models of Information-Seeking Behavior

Performance of the models was mixed and ranged from those with AUROCs above 0.90 to others with AUROCs below 0.50. The mediocre performance of some of the models was likely due to the relatively small sizes of the training data sets and the small number of positive targets. In previous work, we demonstrated that model performance improved as the number of training cases increased, and it is likely that larger training data sets will improve model performance [5]. Moreover, larger training data sets will increase target coverage (ie, the number of targets modeled).

The context of this study was limited to a single clinical task (ICU physicians preparing to present at morning rounds) and two clinical conditions (AKF and ARF). Even in this limited context and in a laboratory setting, there were variations in the information-seeking behaviors of the reviewers leading to differences in annotations for the same patient cases (eg, cases in the evaluation data set were each evaluated by four physicians). These differences partly stem from length of experience and depth of clinical knowledge, with some reviewers in fellowship training with less than two years of experience in critical care and others who are attendings with extensive experience. In active clinical environments, other factors such as urgency, workload, and interruptions will potentially influence the information-seeking behavior, leading to even more variation. In this study, the models were trained using annotations from all reviewers who were weighted equally. However, it may be desirable to either use data from only experienced physicians or weight their data more heavily for training models. Further research is needed to better characterize variability among the information-seeking behavior of physicians.

While prioritizing data can reduce cognitive load, with imperfect model performance, any reduction in cognitive load could be offset by the need to check unprioritized data to ensure that important information is not missed. With improved model performance, a LEMR system could reach high levels of accuracy, leading to a net reduction in cognitive load.

### Limitations and Future Work

We used manual selection as the gold standard in the evaluation data set. This choice provides an advantage to models trained on manual selection training data. Despite the advantage, our results show eye tracking to be a promising, higher-throughput alternative.

The patients included in this study were chosen because they had either AKF or ARF. Future studies are needed to evaluate the effectiveness of machine learning methods when applied to patients with a wider range of clinical problems, comorbidities, and clinical tasks.

The eye-tracking device we used has several limitations, including reduced accuracy at the edges of the monitor and a limited tracking box (the three-dimensional space where a user’s head can be positioned while still capturing coordinates of the user’s eye gaze). To address the device’s limitations, we designed the LEMR user interface to have adequate separation between data items. Further research is needed to determine the performance and limitations of inexpensive eye-tracking devices in an active clinical setting where users are likely to be more active and frequently distracted compared to a laboratory setting. Using LEMR models in conjunction with a commercial EMR poses additional challenges, such as obtaining patient data in real time. One promising approach to explore in the future is to deploy LEMR as a SMART (Substitutable Medical Applications, Reusable Technologies) on FHIR (Fast Healthcare Interoperability Resource) application [27].

### Conclusions

We proposed and evaluated using eye tracking as a novel method to train a LEMR system that prioritizes the display of relevant medical record data. Our results support eye tracking as being a viable method for automatically capturing physician information-seeking behavior. Further studies are needed to evaluate the effectiveness of eye tracking in the clinical environment and to advance it as a practical approach for acquiring large amounts of training data for a LEMR system.

## Abbreviations

AKF: acute kidney failure
ARF: acute respiratory failure
AUROC: area under the receiver operating characteristic curve
EMR: electronic medical record
FHIR: Fast Healthcare Interoperability Resource
ICU: intensive care unit
LEMR: Learning Electronic Medical Record
SMART: Substitutable Medical Applications, Reusable Technologies
